# Risk Interactions of Coronavirus Infection across Age Groups after the Peak of COVID-19 Epidemic

**DOI:** 10.3390/ijerph17145246

**Published:** 2020-07-21

**Authors:** Xinhua Yu

**Affiliations:** Division of Epidemiology, Biostatistics & Environmental Health, School of Public Health, University of Memphis, Memphis, TN 38152, USA; xyu2@memphis.edu; Tel.: +1-901-678-3433

**Keywords:** COVID-19, elderly people, risk interaction, South Korea, virus infection, SARS-CoV-2

## Abstract

Background: The COVID-19 pandemic has incurred significant disease burden worldwide, particularly on the elderly population. This study aims to explore how risks of coronavirus infection interact across age groups using data from South Korea. Methods: Daily new COVID-19 cases from 10 March to 30 April 2020 were scraped from online open sources. A multivariate vector autoregressive model for time series of count data was used to examine the risk interactions across age groups. Case counts from previous days were included as predictors to dynamically examine the change of risk patterns. Results: In South Korea, the risk of coronavirus infection among elderly people was significantly affected by other age groups. An increase in virus infection among people aged 20–39 was associated with a double risk of infection among elderly people. Meanwhile, an increase in virus infection among elderly people was also significantly associated with risks of infection among other age groups. The risks of infection among younger people were relatively unaffected by that of other age groups. Conclusions: Protecting elderly people from coronavirus infection could not only reduce the risk of infection among themselves but also ameliorate the risks of virus infection among other age groups. Such interventions should be effective and for the long term.

## 1. Introduction

Coronavirus disease 2019 (COVID-19) is caused by the infection of a novel severe acute respiratory syndrome-associated coronavirus (SARS-CoV-2) [1,2]. Since December 2019, over 11 million people have been infected with SARS-CoV-2 and over 528,000 people have died of coronavirus infection (https://coronavirus.jhu.edu/map.html, accessed on 4 July 2020). Of them, elderly people and people with underlying chronic conditions suffered the heaviest disease burden [3,4,5]. For example, in the US, as reported by the US Center for Disease Control and Intervention, about 80% of deaths were people aged 65 or above (https://www.cdc.gov/coronavirus/2019-ncov/cases-updates/cases-in-us.html), and 43.4% of hospitalizations were people aged 65 or above [6,7]. On the other hand, the case fatality rate of COVID-19 in South Korea was about 2.1% as of 16 July 2020, according to the Korea Center for Disease Control report (http://ncov.mohw.go.kr/en/).

The reasons for the disproportional burden among elderly people were unclear [8]. Elderly people generally have weaker immune systems than younger people due to aging, and they are also more likely to have multiple chronic conditions [9,10]. Thus, elderly people may have severe symptoms if infected with coronavirus [11,12]. On the other hand, elderly people may have been exposed to myriads of infections over their lifetime which may provide partial immunity against new virus infection. Although cross-reaction of antibodies between SARS-CoV and SARS-CoV-2 was observed, cross-neutralization was rare [13]. Thus, it was unlikely elderly people might have any effective immunity against SARS-CoV-2.

Epidemic data of high quality are essential to understand the mechanism of an epidemic and compare the courses of epidemic and risk patterns of infection across different groups. Unfortunately, due to insufficient testing kits, heterogeneous diagnosis criteria, and varied implementation of different interventions, epidemiological data on COVID-19 pandemic among different countries (and even within a country) and during different periods were often noncomparable [14]. One notable exception is South Korea, where extensive contact tracing and mass testing not only curtailed the epidemic but also generated high-quality data. In South Korea, both asymptomatic and symptomatic cases were identified promptly from the very beginning of the epidemic [15,16]. Thus, depicting a complete picture of the epidemic process was possible in South Korea.

**Figure 1:**
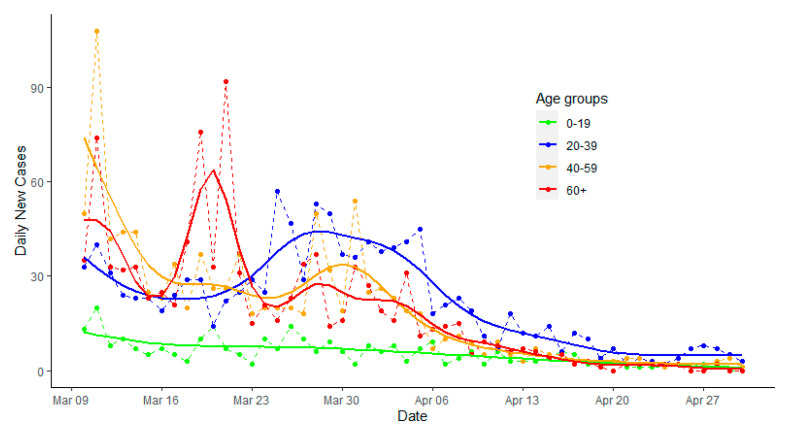
Observed daily new cases of COVID-19 and predicted epidemic curve from the generalized additive model, South Korea, from 10 March to 30 April 2020, by age groups.

In South Korea, the first case of COVID-19 occurred on 20 January 2020 and the major outbreak started on 19 February 2020. The COVID-19 pandemic was waning down since 10 March 2020 (see Figure 1) [17,18]. However, potential rebounds of new cases have been warned by many public health experts [19]. This is reflected in an epidemic curve with a long tail and occasional spikes, which is demonstrated in the epidemic process in South Korea (https://www.kcdc.info/covid-19/) [15,16]. In addition, if the seasonality, immunity, and cross-immunity of SARS-CoV-2 behave like previous coronaviruses, a recent study predicted that a long-lasting and multi-wave epidemic was possible [20]. Furthermore, after the first epidemic peak is passed, which suggests mitigating interventions are likely effective, the society is expected to gradually return to normalcy [21]. Strategies for re-opening the society should be evaluated, and exploring risk patterns of infection during the post-peak period will provide important evidence for decision-making. In the COVID-19 epidemic, given the disproportional disease burden born by elderly people, it is imperative to examine risk patterns of coronavirus infection among elderly people after the peak of the epidemic. Epidemic data from South Korea will allow us to examine the risk interactions among different age groups, which can serve as an exemplary reference model for other countries. 

In this study, we will examine how risks of coronavirus infection interact across age groups using time series analysis. Using the high-quality data from South Korea, we will focus on the post-peak period of the epidemic process to evaluate the risk of infection among elderly people during the period of society re-opening. 

## 2. Materials and Methods 

Daily new COVID-19 case counts from South Korea were obtained from the website (https://www.kaggle.com/kimjihoo/coronavirusdataset) which were scraped from the Korea Center for Disease Control website. The first COVID-19 case in South Korea appeared on 20 January 2020, and the major epidemic started on 19 February 2020. Since the peak of the first major epidemic wave in South Korea ended around 10 March [22] (also Figure 1), we limited the time series of new cases from 10 March to 30 April 2020. All daily cases were stratified by age groups (0–19, 20–39, 40–59, and 60 or above). Those aged 60 or above were referred to as elderly people.

The observed epidemic curves by age groups from 10 March to 30 April 2020 were plotted, and the predicted daily cases were obtained from a generalized additive model (GAM), [23] assuming daily new cases follow Poisson distributions. The smoothness of predicted values was achieved with thin plate regression splines with 16 knots using the R *mgcv* package (see Appendix A). The number of knots was determined empirically in which 16 knots best captured the non-linearity of the epidemic curve without being too smooth and too rough.

We developed a vector autoregressive (VAR) model to examine the associations of the infection risks across age groups simultaneously [24]. Specifically, we assumed daily new case counts (y_j,t_) followed a generalized Poisson distribution to account for over-dispersion of case counts (i.e., observed variance is larger than expected variance) [25]. The model also included case counts from previous days (lags) across age groups as predictors to form a dynamic model (see Appendix A for details). Therefore, the current risk of infection in each age group was predicted not only by previous case counts in its own group but also by previous counts from other age groups.

(1) $$\begin{array}{c} y_{j,t}\;|\alpha_{j},\beta_{j,k},b_{j,t}\sim \;generalized\;Poisson\left(\mu_{j,t},\xi_{j}\right)\\ \mu_{j,t}=\exp\left(\alpha_{j}+\sum_{j=1}^{J}\sum_{k=1}^{K}\beta_{j,k}\ln(y_{j,t-k})+b_{j,t}\right)\\ b_{j,\;t\;}|\Sigma \;\sim \;MultiNormal\left(0,\Sigma \right) \end{array}$$

where *j* = 1, …, *J* represents age groups, *t* = 1, …, *T* represents days, and *k* = 1, …, *K* represents the number of time lags. Since the typical incubation period of COVID-19 is five days [26], we reported results from five-lag models, that is, the daily counts from one prior day (yesterday), two prior days (the day before yesterday), and from up till five days before were included as predictors in the models. Three-lag and seven-lag models were also explored, and results from all models were consistent (see online codes and results). The scale parameter ξ in the generalized Poisson distribution controls the magnitude of dispersion, that is, ξ = 0 corresponding to a standard Poisson (mean = variance), ξ < 0 suggesting under-dispersion (mean > variance), and 0 < ξ < 1 indicating overdispersion (mean < variance). The *b_j,t_* could be viewed as a random effect to account for the correlation of daily counts between age groups. The *b_j,t_* was assumed a multivariate normal distribution.

The above model framework was similar to the common log-linear relative risk models in epidemiological studies which assume multiplicative associations between predictors and outcomes [27]. The coefficients βs could be interpreted as natural logarithms of risk ratios per one-unit change of natural logarithms of case counts.

We fit the above models with the Bayesian software stan through the Rstan interface (http://mc-stan.org) [28]. To keep the model simple, we assumed weakly informative priors of student t distributions for all αs and βs, and an LKJ prior with modal density around diagonals for correlations between case series (see Appendix A). Hamiltonian Monte Carlo was used to obtain posterior distributions of parameters. Diagnostic plots showed all chains mixed satisfactorily and were converged. In addition, negative binomial models were also fitted, and results were similar to those reported here except for wider confidence intervals (Appendix B, Table A1). Notice that the dispersion factors (1/ξ) estimated from the generalized Poisson models were 2.66 (1.58–5.13), 1.44 (0.96–2.46), 2.21 (1.37–3.82), and 0.90 (0.59–1.50) for those aged 60 or above, 40–59, 20–39, and 0–19, respectively. These estimates were of moderate magnitude and two of them (age group 40–59 and 0–19) were not statistically different from 1. This also suggested that negative binomial models might overestimate the dispersion factors which led to wider confidence intervals. The data, replicable codes, and other results are available online (www.github.com/xinhuayu/riskinteractions/).

*Ethics statement:* This study was based on publicly available data. There was no direct involvement of human subjects. Therefore, it was exempted from the approval of the institutional review board. No informed consent was needed. All authors declared no conflict of interest in conducting this study.

## 3. Results

In South Korea, there were 3383 COVID-19 cases between 10 March and 30 April 2020. Of them, 283 cases were people aged 0–19 (8.4%), 1141 aged 20–39 (50.0%), 987 aged 40–59 (29.2%), and 972 aged 60 or above (28.7%). 

Figure 1 presents the epidemic curves with fitted values by age groups for South Korea. After 10 March, there was a small spike among those aged 60 or above around 20 March 2020, and a small rebound among those aged 20–39 (e.g., around 30 March to 5 April 2020), followed by those aged 40–59 and aged 60 or older.

Table 1 describes associations of risks of infection across age groups in South Korea. In addition to tracking the effect from the first lag day (yesterday) among elderly people, the current daily counts of infection among elderly people were doubled (risk ratio = 2) with a one-unit increase in the natural logarithm of case counts (or 2.8 times increase in daily counts) on the day before yesterday (lag 2) among those aged 20–39. Additionally, an increase in infection during the fourth prior day (lag 4) in those aged 20–39 was associated with a decrease in daily counts in elderly people, possibly due to a periodic effect. As the epidemic came in waves, an increase in the case counts in the young age group would have taken an additional four days to qualitatively impact the risk of infection among elderly people. Finally, an increase in infection in the youngest population during the fifth lag days was also associated with an increased risk of infection among elderly people by 64%.

More importantly, an increase in virus infection among elderly people was associated with increases in risks of infection among all other age groups, but with longer delays in younger populations. Furthermore, the risk of infection among people aged 40–59 was affected by both old and young people, but to a lesser extent. Risks of infection among people aged 20–39 or 0–19 were less likely affected by other age groups.

## 4. Discussion

This was the first study to quantify risk interactions of SARS-CoV-2 infection across age groups based on vector autoregressive models using epidemic data of high quality from South Korea. We found that in South Korea, the risk of infection among elderly people was significantly affected by other age groups. An increase in virus infection among elderly people was also significantly associated with increased risks of infection among other age groups. Risks of infections among younger people were relatively unaffected by that of other age groups.

Our results were consistent with the current COVID-19 epidemic process, in which risk of infections among elderly people might be affected by other age groups [8]. Although virus transmission might differ among age groups [8,29], the risk interactions were likely due to personal interactions between people of different age groups. Respiratory infectious diseases often spread through personal contacts [30]. Previous studies showed that contacts were more frequent in young age groups than older age groups, and interactions across age groups were less frequent than within each age group [31]. During the emerging pandemic of COVID-19, stringent control measures such as lock-down, strict social distancing, and stay-at-home rules, were often implemented promptly, leading to an abrupt and significant change in contact patterns within and between age groups. Modern techniques such as contract tracing apps, infection risk IDs, and instant notifications of cases also allowed us to efficiently isolate cases and quarantine high-risk people. The observed risk interactions between age groups in South Korea might be largely due to the change in contact patterns during the pandemic period. As shown in our study, there were two–five lagging days in the risk interactions across age groups, especially between old and young people. On the other hand, the infection among elderly people may still be affected by and also affect the risks of infection among other age groups. In addition, in the US, half of the infected cases could not be traced back to known cases in a telephone survey conducted in outpatient and inpatient settings of academic medical centers [32]. Therefore, although elderly people may refrain from contacting known cases, they may be still at risk of infection by doing regular activities such as grocery shopping. Passive community interactions may play an important role in sustaining the epidemic.

Our results highlighted the importance of implementing and enforcing effective interventions on the whole society [33,34,35], and the higher priority of protecting elderly people [29]. Furthermore, we showed that an increase in coronavirus infection among elderly people was associated with increased risks of infection among other age groups, suggesting protecting elderly people and reducing the risk of infection among elderly people had a spillover effect on the whole society. This was consistent with our previous simulation study in which reducing contacts among the elderly could reduce the virus infection and hospitalizations in the whole society [36].

There were some limitations in this study. The most important one was that we relied on reported cases. The data from South Korea were more likely complete due to extensive contact tracing and mass testing. Furthermore, the case reporting date (or virus infection detection/lab confirmation date) was different from the virus infection date, and the average incubation period for SARS-CoV-2 was about five days [26]. The laudable efforts of extensive contact tracing and mass testing implemented by the South Korea government since the beginning of COVID-19 epidemic significantly reduced reporting delays, and likely identified many cases before symptom onsets [15]. Therefore, the interval between virus infection and case reporting might be small. In addition, there were other factors such as gender, socio-economic status, and neighborhood environment which might have also affected the risk of infection.

Moreover, although we interpreted the results with action terms, they had no explicit causative meanings. For example, younger people tended to have milder or no symptoms (i.e., subclinical cases) if infected with the coronavirus [37,38,39]. Thus, it was possible that an increased number of detected cases among young people implied the existence of an increase in subclinical cases in the community who might unknowingly infect other people, including elderly people. Subclinical cases could only be identified through extensive contact tracing and mass testing. Without this information, it is impossible to examine the route of infections in the community.

Our study has several strengths. Firstly, data from South Korea were more likely complete which provided instrumental information about underlying epidemic mechanisms. Although different social norms and health care systems might explain some differences in risk patterns between South Korea and other regions, results from South Korea provided a baseline picture of risk interactions among age groups under a well-controlled, ideal epidemic process. Different patterns might be due to differences in population structures, magnitudes of control measures, and contact patterns in the society, while similar patterns in risk interactions between regions allowed us to infer the possible paths of infections.

Secondly, we proposed a novel multivariate autoregressive model for time series of counts to examine the risk of virus infection across age groups simultaneously. A flexible generalized Poisson model fitted with Bayesian methods was used to account for overdispersion of count data [25]. Unlike many other studies that used mechanistic epidemic models which were useful to describe the epidemic process [40], our statistical models extended traditional relative risk models to time series of count data. It should be noted that this type of model likely overfits the data, and collinearity among lag variables also exists. Thus, having a priori hypotheses and choosing biologically relevant lags are critical in building correct models and interpreting the results. Our lag models were based on the observed incubation period of COVID-19 and for testing pre-specified hypotheses. The principle of our methods was similar to that of the Institute for Health Metrics and Evaluation (IHME) [41] and University of Texas-Austin models [42], all of which relied on time series analysis of count data. However, we did not attempt to predict future cases. Rather, we focused on disentangling risk interactions of infection across age groups, which is more important and relevant in disease prevention.

Finally, during the process of re-opening the economy and society, the number of new cases may rebound, and multiple small waves or a second big wave of epidemic are possible. A contentious issue was whether and how to protect high-risk populations such as elderly people during the return of the epidemic. Therefore, we limited our study period to the post-peak of the epidemic to answer this imminent question. Our study strongly supported that high-risk populations such as elderly people should still take serious precautions during the post-epidemic period.

## 5. Conclusions

In summary, protecting elderly people from coronavirus infection might not only be associated with a reduced risk of infection among themselves but also related to lower risks of virus infection among other age groups. Therefore, elderly people should keep on practicing social distancing and maintaining effective personal protection while still maintaining a good care of their comorbidities.

## Figures and Tables

**Table 1:** Risk ratios in the coronavirus infection across age groups during the COVID-19 pandemic, South Korea, 10 March to 30 April 2020.

Model Outcomes	Predictors	Lags of Predictors				
		Lag 1	Lag 2	Lag 3	Lag 4	Lag 5
Aged 60 or above						
	60 or above	2.09 (1.28–3.17) *	1.21 (0.78–1.80)	0.90 (0.60–1.39)	1.03 (0.60–1.67)	0.96 (0.63–1.53)
	40–59	0.95 (0.49–1.84)	1.81 (0.98–3.29)	0.59 (0.30–1.18)	1.37 (0.85–2.23)	1.03 (0.61–1.70)
	20–39	0.89 (0.53–1.46)	2.02 (1.12–3.47) *	1.28 (0.72–2.31)	0.41 (0.22–0.78) #	1.11 (0.58–2.22)
	0–19	0.90 (0.61–1.43)	1.36 (0.95–1.95)	0.82 (0.57–1.19)	0.61 (0.44–0.85) #	1.64 (1.03–2.58) *
Aged 40–59						
	60 or above	1.66 (1.19–2.29) *	0.79 (0.55–1.12)	0.89 (0.64–1.23)	0.98 (0.70–1.40)	1.02 (0.71–1.40)
	40–59	1.01 (0.62–1.63)	1.76 (1.16–2.66) *	1.23 (0.76–1.96)	0.89 (0.62–1.29)	0.88 (0.62–1.28)
	20–39	1.13 (0.75–1.73)	1.12 (0.76–1.67)	1.59 (1.03–2.50) *	0.60 (0.37–0.95)	0.88 (0.53–1.48)
	0–19	1.04 (0.79–1.37)	1.28 (0.97–1.68)	1.17 (0.88–1.57)	1.14 (0.88–1.49)	1.17 (0.84–1.62)
Aged 20–39						
	60 or above	0.95 (0.69–1.29)	0.89 (0.65–1.23)	1.01 (0.72–1.39)	1.54 (1.11–2.12) *	0.97 (0.69–1.31)
	40–59	1.17 (0.73–1.88)	1.18 (0.78–1.78)	0.98 (0.60–1.52)	0.90 (0.64–1.26)	1.04 (0.75–1.47)
	20–39	1.56 (1.05–2.36) *	1.02 (0.68–1.55)	1.45 (0.96–2.24)	1.06 (0.68–1.64)	0.85 (0.55–1.32)
	0–19	1.04 (0.79–1.37)	0.96 (0.75–1.27)	0.88 (0.66–1.20)	0.97 (0.75–1.26)	0.74 (0.54–1.00)
Aged 0–19						
	60 or above	1.78 (1.23–2.61) *	1.34 (0.86–2.06)	0.99 (0.67–1.51)	0.82 (0.52–1.25)	1.55 (1.02–2.30) *
	40–59	0.76 (0.41–1.38)	0.71 (0.42–1.18)	0.85 (0.47–1.56)	0.91 (0.55–1.57)	0.97 (0.62–1.54)
	20–39	1.51 (0.92–2.55)	1.50 (0.85–2.57)	1.04 (0.61–1.80)	0.78 (0.44–1.38)	0.91 (0.51–1.62)
	0–19	0.93 (0.64–1.36)	0.85 (0.60–1.21)	0.85 (0.60–1.27)	0.92 (0.62–1.38)	1.51 (0.99–2.31)

Note: * and # for *p* < 0.05. Lag 1 of predictors means daily counts from previous day (yesterday), lag 2 means daily counts from the day before yesterday and so forth.

## Abbreviations

The following abbreviations are used in this manuscript:

SARS-CoV-2	severe acute respiratory syndrome coronavirus 2
COVID-19	coronavirus infectious disease 2019
GAM	generalized additive model
VAR	vector autoregressive regression
NB	negative binomial model
REML	restricted maximum likelihood
PMF	probability mass function

## Appendix A

### Appendix A.1. Obtained Smoothed Predicted Daily Cases with Generalized Additive Model (GAM)

Assuming daily new cases follow a Poisson distribution or negative binomial (NB) distribution (see below), the GAM is a linear regression with a smoothed time term. For simplicity, a separate GAM regression was fitted for each age group:

(A1) $$\log_{e}\left(E\left(Y_{ij}\right)\right)=\beta_{0}+\overset{K}{\underset{k=1}{\sum }}\beta_{j,k}\cdot b_{k}\left(time_{i}\right)$$

where *Y_ij_* represents the observed case counts of day *i* and group *j*, and *E*(*Y_ij_*) is the expected (predicted) value. The variable *time_i_* represents day (1,…,I), *b_k_*( ) represents a basis function for the *k*th term to the smooth temporal trend, and *β_j,k_s* are regression coefficients for the smooth term *k* and group *j*. The restricted maximum likelihood (REML) approach was used in the parameter estimation. The R *mgcv* package was used [23], and smooth terms were fitted using a thin plate regression spline with 16 knots. The number of knots is typically determined empirically, commonly with 8–20 knots. A smaller number of knots yields a smoother curve while a larger number of knows results in a rougher curve. We observed that 16 knots are reasonable to capture the nonlinearity of the epidemic curve without introducing too many spikes.

### Appendix A.2. Model Setups and Comparisons

The standard Poisson distribution describes the distribution of y events occurring at a constant rate of λ. The probability mass function (PMF) is

(A2) $$\begin{array}{c} p\left(Y=y|\lambda \right)=\frac{\lambda^{y}}{y!}e^{-\lambda }\;,\;\lambda >0\\ E\left(Y\right)\;≝\;\mu =\lambda =Var\left(Y\right)\\ Loglik=ylog\left(\lambda \right)-\lambda -log\left(y!\right) \end{array}$$

In standard Poisson distribution, expected variance equals the mean. If observed variance is larger than expected variance (i.e., the mean), then overdispersion exists. This often occurs when outcomes are correlated, such as daily new case counts during a disease outbreak.

The generalized Poisson distribution introduces an additional scale parameter **ξ** [43] as quoted in Hilbe JM. 2014 [27]. The PMF is

(A3) $$\begin{array}{c} p\left(Y=y|\lambda ,\xi \right)=\frac{\lambda }{y!}{\left(\lambda +\xi y\right)}^{y-1}e^{-\left(\lambda +\xi y\right)},\;\lambda >0\\ E\left(Y\right)\;≝\;\mu =\frac{\lambda }{1-\xi }\\ Var\left(Y\right)=\frac{\lambda }{{\left(1-\xi \right)}^{3}}=\frac{\mu }{{\left(1-\xi \right)}^{2}} \end{array}$$

Thus, $\varphi \;≝\;\frac{1}{{\left(1-\xi \right)}^{2}}$ is the dispersion factor indicating how variance changes with the mean. Therefore, if ξ = 0, then φ = 1 corresponds to a standard Poisson (mean = variance); 0 < ξ < 1, then φ > 1 models overdispersion (mean < variance); and ξ < 0, then φ < 1 models under-dispersion (mean > variance).

Reparametrize the PMF of generalized Poisson distribution with μ and ξ [25]:

(A4) $$\begin{array}{c} p\left(Y=y|\mu ,\xi \right)=\frac{\mu \left(1-\xi \right)}{y!}{\left(\mu -\xi \left(\mu -y\right)\right)}^{y-1}e^{-\left(\mu -\xi (\mu -y\right))}\\ LogLik=log\left(\mu \left(1-\xi \right)\right)+\left(y-1\right)log\left(\mu -\xi \left(\mu -y\right)\right)-\left(\mu -\xi \left(\mu -y\right)\right)-\log\left(y!\right) \end{array}$$

On the other hand, the negative binomial distribution describes the distribution of the number of successes given a predefined r number of failures during a sequence of independent Bernoulli trials with a success probability p:

(A5) $$\begin{array}{c} p\left(Y=y\right)=\left(\begin{array}{c} y+r-1\\ y \end{array}\right)p^{y}{\left(1-p\right)}^{r}\\ E\left(Y\right)\;≝\;\mu =\frac{rp}{1-p}\\ Var\left(Y\right)=\frac{rp}{{\left(1-p\right)}^{2}}=\frac{\mu }{1-p}=\mu +\frac{\mu^{2}}{r} \end{array}$$

Thus, the overdispersion of Y is controlled by the shape parameter r.

Reparametrize the PMF with μ and r:

(A6) $$p\left(Y=y\right)=\left(\begin{array}{c} y+r-1\\ y \end{array}\right){\left(\frac{\mu }{\mu +r}\right)}^{y}{\left(\frac{r}{\mu +r}\right)}^{r}$$

(A7) Loglik=yr(log(μr)−2log(μ+r))+log(Γ(y+r))−log(Γ(y+1))−log(Γ(r))

where gamma function Γ(x+1) = x! (x factorial) is for an integer x, and the parameter r can be any positive real value.

Note that negative binomial distribution can be viewed as a gamma–Poisson mixture distribution in which Y ~ Poisson(λ) and λ ~ gamma(*r, λ/r*). That is, the negative binomial distribution (r, p) is the posterior distribution of Poisson(λ) with gamma(*r, λ/r*) as the conjugate prior of λ, where λ ≝ μ=rp/(1−p). Rewriting gamma(*r, λ/r*) as gamma(*r*, p/(1−p)) and using Γ functions to represent factorials:

(A8) $$\int_{0}^{\infty }\frac{\lambda^{y}e^{-\lambda }}{\Gamma \left(y+1\right)}\cdot \frac{\lambda^{r-1}e^{-\lambda \left(\frac{1-p}{p}\right)}}{{\left(\frac{p}{1-p}\right)}^{r}\Gamma \left(r\right)}d\lambda =\frac{\Gamma \left(r+y\right)}{\Gamma \left(r\right)\Gamma \left(y+1\right)}p^{y}{\left(1-p\right)}^{r}$$

Under this framework, negative binomial distribution is appealing as a natural extension of Poisson distribution to allow for overdispersion that is controlled by the shape parameter *r*.

However, although negative binomial distribution is often used to model new case counts during disease outbreaks, it models only overdispersion and assumes a quadratic relationship between variance and mean, while the generalized Poisson model is more flexible and assumes a simpler first order association between variance and mean. Therefore, we chose to report results from generalized Poisson models. Results from negative binomial models are included in the Appendix B. In addition, it is also of note that there are extensions of negative binomial models in which the association between mean and variance can be estimated from data, leading to a more flexible model and also permitting the exploration of determinants of overdispersion [27].

In this study, we proposed the following hierarchical vector autoregressive model (VAR) for count data:

(A9) $$\begin{array}{l} y_{j,t}\;|\alpha_{j},\beta_{j,k},b_{j,t}\sim \;PMF\left(\mu_{j,t}\right)\\ \mu_{j,t}=\exp\left(\alpha_{j}+\sum_{j=1}^{J}\sum_{k=1}^{K}\beta_{j,k}\ln(y_{j,t-k})+b_{j,t}\right)\\ b_{j,t\;}|\Sigma \;\sim \;MultiNormal\left(0,\Sigma \right) \end{array}$$

where *j* = 1, …, *J* represents age groups, *t* = 1, …, *T* represents days, and *k* = 1, …, *K* represents the number of lags. The PMF of Y can be either standard Poisson (λ), generalized Poisson (μ, ξ), or negative binomial (μ, *r*) distribution.

The above VAR model included new case counts from previous days (lags) across age groups as predictors [24], thus examining associations of the infection risks across age groups simultaneously. That is, the current risk of infection in each age group was predicted not only by previous case counts in its own group but also by previous counts from other age groups.

The correlation of daily counts between age groups was modeled through *b_j,t_* that can be viewed as a random effect. The *b_j,t_* was assumed a multivariate normal distribution.

The exponential link between dynamic predictors and μ is equivalent to common relative risk models in epidemiological studies, i.e., log-linear models for count data. Under this multiplicative scale framework, the interpretations of βs are relative risks given a one-unit increase in predictors.

During the model fitting, we assumed some weakly informative priors for all parameters:

(A10) $$\begin{array}{c} \alpha_{j}\sim t\left(df=5,location=0,scale=2.5\right)\\ \beta_{j,k}\sim t\left(df=5,location=0,scale=2.5\right)\\ \xi_{j}\sim normal\left(0,0.3\right),\;and-0.9\leq \xi_{j}\leq 0.9\\ \frac{1}{\sqrt{r}}\;≝\;\;\varphi \;\sim \;\mathrm{positive}\;\mathrm{half}-\mathrm{normal}\left(0,1\right)\\ \Sigma \;=D^{\prime }RD,\;\\ \;where\;standard\;deviation\;D=diag\left(sd_{j}\right),\;sd_{j}\sim cauchy\left(0,5\right)\\ \;and\;R=correlation\;matrix,R\sim \;LKJ\left(2\right) \end{array}$$

The LKJ prior is a special prior most suitable for correlations. The LKJ(2) assumes a modal density surrounding diagonals.

The models were fit with Bayesian software stan through the Rstan interface [28]. A customized stan function was constructed for fitting the generalized Poisson model. We employed Hamiltonian Monte Carlo with five Markov chains, each with 50,000 iterations plus 2000 warmups, to obtain posterior distributions of parameters. Diagnostic plots through the shinestan package showed all chains mixed well and were converged. The replicable data and codes, including models with daily case counts as standard Poisson, generalized Poisson, or negative binomial distributions, are available online (www.github.com/xinhuayu/riskinteractions/).

## Appendix B. Additional Table

**Table A1:** Risk interactions in coronavirus infection across age groups based on negative binomial models, COVID-19, South Korea.

Model Outcomes	Predictors	Lags of Predictors				
		Lag 1	Lag 2	Lag 3	Lag 4	Lag 5
Aged 60 or above						
	60 or above	1.93 (1.14–3.19) *	1.09 (0.66–1.80)	1.02 (0.61–1.82)	0.99 (0.57–1.67)	1.11 (0.68–1.87)
	40–59	1.03 (0.53–2.02)	1.66 (0.83–3.01)	0.69 (0.36–1.46)	1.26 (0.72–2.25)	1.00 (0.57–1.71)
	20–39	0.94 (0.54–1.69)	1.30 (0.66–2.42)	1.42 (0.73–2.68)	0.50 (0.25–1.01)	1.21 (0.63–2.37)
	0–19	1.10 (0.72–1.78)	1.29 (0.79–1.98)	0.85 (0.51–1.35)	0.62 (0.40–0.98) #	1.52 (0.88–2.55)
Aged 40–59						
	60 or above	1.64 (1.12–2.41) *	0.75 (0.50–1.09)	0.97 (0.67–1.49)	1.01 (0.69–1.53)	1.02 (0.69–1.53)
	40–59	1.15 (0.68–1.96)	1.55 (0.92–2.46)	1.14 (0.64–2.24)	0.90 (0.54–1.39)	0.95 (0.63–1.47)
	20–39	1.10 (0.59–1.73)	1.13 (0.70–1.81)	1.54 (0.95–2.47)	0.63 (0.37–1.27)	0.89 (0.44–1.56)
	0–19	1.09 (0.77–1.53)	1.23 (0.87–1.78)	1.16 (0.82–1.64)	1.16 (0.82–1.60)	1.15 (0.79–1.68)
Aged 20–39						
	60 or above	1.00 (0.71–1.44)	0.93 (0.65–1.39)	1.00 (0.69–1.48)	1.58 (1.10–2.26) *	0.90 (0.64–1.29)
	40–59	1.12 (0.69–1.86)	1.09 (0.67–1.72)	0.96 (0.58–1.53)	0.97 (0.65–1.44)	1.08 (0.72–1.61)
	20–39	1.63 (1.09–2.48) *	1.04 (0.66–1.64)	1.35 (0.86–2.11)	0.99 (0.58–1.56)	0.96 (0.60–1.56)
	0–19	1.04 (0.74–1.44)	0.95 (0.69–1.29)	0.89 (0.63–1.23)	0.95 (0.67–1.40)	0.73 (0.51–1.04)
Aged 0–19						
	60 or above	1.77 (1.17–2.76) *	1.35 (0.82–2.14)	1.00 (0.62–1.61)	0.81 (0.50–1.32)	1.52 (0.97–2.44)
	40–59	0.76 (0.39–1.42)	0.71 (0.39–1.27)	0.86 (0.43–1.64)	0.94 (0.53–1.66)	0.96 (0.58–1.60)
	20–39	1.53 (0.87–2.72)	1.46 (0.82–2.69)	1.05 (0.57–1.92)	0.78 (0.41–1.41)	0.90 (0.49–1.74)
	0–19	0.94 (0.63–1.44)	0.83 (0.57–1.25)	0.88 (0.58–1.33)	0.92 (0.59–1.44)	1.52 (0.95–2.42)

Note: * and # for *p* < 0.05.
